# Performance and Transient Behavior of Vertically Integrated Thin-film Silicon Sensors

**DOI:** 10.3390/s8084656

**Published:** 2008-08-08

**Authors:** Nicolas Wyrsch, Gregory Choong, Clément Miazza, Christophe Ballif

**Affiliations:** Institut de Microtechnique (IMT), University of Neuchâtel, Breguet 2, 2000 Neuchâtel, Switzerland E-mails: nicolas.wyrsch@unine.ch; gregory.choong@unine.ch; christophe.ballif@unine.ch.

**Keywords:** Image sensor, monolithic integration, amorphous silicon

## Abstract

Vertical integration of amorphous hydrogenated silicon diodes on CMOS readout chips offers several advantages compared to standard CMOS imagers in terms of sensitivity, dynamic range and dark current while at the same time introducing some undesired transient effects leading to image lag. Performance of such sensors is here reported and their transient behaviour is analysed and compared to the one of corresponding amorphous silicon test diodes deposited on glass. The measurements are further compared to simulations for a deeper investigation. The long time constant observed in dark or photocurrent decay is found to be rather independent of the density of defects present in the intrinsic layer of the amorphous silicon diode.

## 1. Introduction

Active pixel sensors (APS) in CMOS technology have recently gained a lot of interest. Many functionalities can be implemented at the pixel level, ranging from basic charge integration, amplification to pre-processing of the data. However, the fact that the pixel readout electronics shares the die area with the sensor elements may limit considerably the sensitivity, and also leads to the presence of dead areas (between the photodiodes) which is unacceptable for certain applications. The reduction of feature size in CMOS technology renders the problem more acute because the sensor area is further reduced and the increase in the number of the metal layers introduces difficulties for the coupling of light with the sensor.

Vertical integration of hydrogenated amorphous silicon (a-Si:H) sensors on top of readout electronics is a promising solution to this problem. This concept has been pioneered by the University of Siegen [1] with the demonstration of this technology for several applications, especially for vision sensors with high sensitivity [2,3] or high dynamic range [4]. This integration concept is known as thin-film on ASIC (TFA), thin-film on CMOS (TFC), above IC (integrated circuit) or elevated diode technology (in the cases where a diode is used) and has attracted a large interest, not only for imaging application but also for particle detection, MEMS (micro electro-mechanical systems) or BioMEMS (biological MEMS) [5].

For light detection, TFA technology offers several advantages compared to c-Si technology with embedded photodiodes:

- Maximization of sensitivity since the entire chip area may be dedicated to light collection.
- Separation between optimization of the photodiode and design of the CMOS circuit.
- Flexibility in the choice of the active material for the photodiode allowing the adjustment of the spectral sensitivity.
- Vertical integration of several photodiodes forming a multi-junction device or combination with other functional layers such as a scintillating layer for X-ray to light conversion

**Figure 1.**
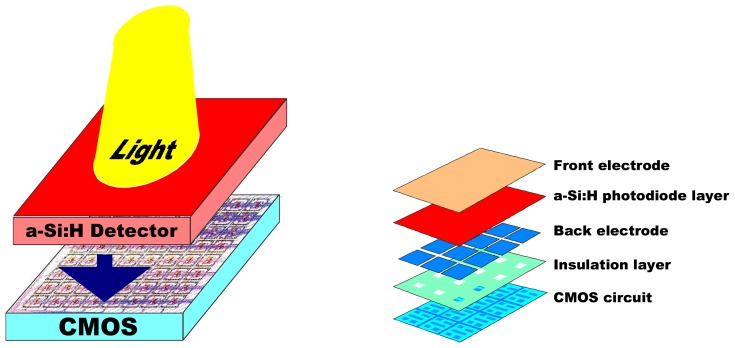
Schematic view of an array of sensors in TFA technology. In most cases, the CMOS circuit passivation layer is used as the insulation layer. The top metal layer of the CMOS chip is either used as the back electrode of the a Si:H diode layer or an additional metal layer is evaporated on top of the chip. The individual pixels pf the array are defined by the patterning of the back electrode.

The typical structure of such a device is presented in Fig. 1.

In this context, a-Si:H offers two significant advantages: (a) low deposition temperature (around 200°C) which the direct deposition of a-Si:H on CMOS chips, and (b) a larger band gap (larger than that of crystalline silicon), leading to a low dark current.

a-Si:H diodes have been optimized at IMT Neuchâtel for the fabrication of TFA sensors for visible light [6], X-ray and particle sensing [7]. In this context, diodes with dark current *J_dark_* as low as 1 pA/cm^2^ and corresponding TFA sensors with *J_dark_* of 12 pA/cm^2^ (both at bias voltage of -1 V) have been fabricated [6]. The issues regarding the design of a-Si:H photodiodes and specifically the influence of the CMOS chip design/topology on the performances of the a-Si:H photodiodes have already been discussed in details [6, 8].

**Figure 2.**
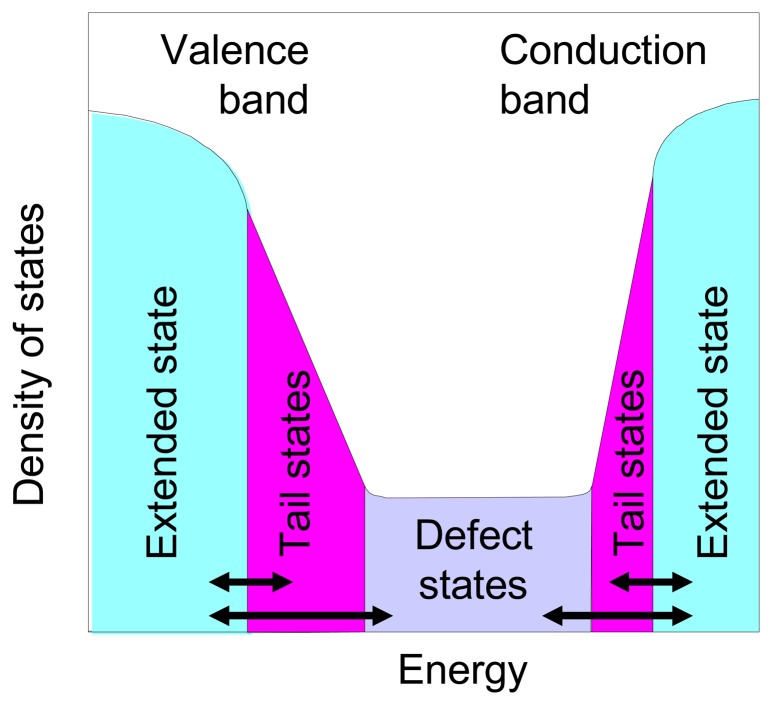
Schematic band diagram of a-Si:H. The continuous state distribution in the pseudo gap, tail states and defect states, is acting as charge reservoir which can be filled-up and emptied during operation of a-Si:H photodiodes and is controlling the transient behavior of the device.

In this paper, we will focus on the performance of TFA image sensors and will analyze the transient behavior of a-Si:H diodes. a-Si:H exhibits a continuous distribution of localized states in the band gap (more exactly of the pseudo gap, see Fig. 2). This distribution comprises tails states due to the disorder present in the amorphous silicon and defect states due to Si dangling bonds. Any change in the polarization or of the illumination level of an a-Si:H diode will perturb the equilibrium between free carriers in the band and trapped carriers in the localized states leading to transient behavior of such device. The objective of this paper is to analyze those transients in test diodes and corresponding TFA imagers.

Effect of carrier trapping and release in a-Si:H diode has already been investigated in previous studies and modeled by simple Shockley-Read statistics [9,10]. The present work focuses on the photocurrent decay kinetics of state-of-the-art a-Si:H diodes in TFA sensors, including simulations using a full description of a-Si:H state distribution.

## 2. Experimental details

Several imagers in using TFA technologies were fabricated by depositing (0.5-2 μm thick) a-Si:H diode arrays both in the metal-i-p and in the n-i-p configurations on standard passivated CMOS chips as well as unpassivated ones covered with a common top 65 nm thick ITO electrode. These chips consisted in an array of 64×64 pixels, with a pixel lateral size of 33 μm (passivated chip) or 38.4 μm (unpassivated) and a pitch of 40 μm from Alcatel-Mitag 0.5 μm MPW (multiple project wafer) technology. For half of the chips, pixels were connected within the CMOS chip to an individual charge integrator while the other half was used to test other internal circuit designs and was not available for imaging. A fill factor of ≈92% was achieved for the imager on unpassivated chips.

Corresponding large-area devices (test diodes) were also deposited on Cr coated glass for the optimization of the a-Si:H diodes deposited on CMOS readout chips as well as for the analysis of some of their transient behavior. In contrast to a-Si:H devices integrated on CMOS chips, individual diode area was defined by patterning the top 65 nm thick ITO layer.

All a-Si:H devices were deposited using Very High Frequency Plasma Enhanced Chemical Vapor Deposition (VHF PECVD), details of diode fabrication have been presented elsewhere [6, 11]. For the test structures on glass, 1 μm thick diodes were deposited on 200 nm chromium coated glass and the pixel areas were defined by patterning the top 65 nm thick transparent conductive oxide contact (ITO). Due to the use of chips from MPW, all processing steps for the fabrication of TFA imagers were done on single chips.

Various setups were used for the monitoring of the transient photocurrent caused by a light pulse from a red LED (λ=650 nm, 3.2×10^13^ photons cm^-2^s^-1^). All the measurements were made in the dark (no bias light) at room temperature. Steady-state dark current for different voltages, as well as slow photocurrent decay were measured using a Keithley 6517 electrometer and current values were measured every 1 s. A delay of 10 minutes was applied between the setup of the bias voltage and the start of the light pulse and data acquisition in order to reach a steady state for the dark current. The slow decay of the photocurrent after the switch off of the light was then measured for different bias voltage. The same procedure was used with the TFC image sensor, and the integration time of the charge integrator was varied from 20 μs to 3 s, in order to measure the illuminated state as well as the steady-state dark current. Light soaking degradation was performed at 50°C under an illumination of 1 sun in an open circuit configuration.

For the fast current transient monitoring, a setup using a transimpedance amplifier (OPA627) with a transfer ratio k=V_OUT_/I_IN_ of 108 V/mA was designed. In this configuration, the photocurrent for single light pulse, as well as periodic light pulse, and the rise and the fall time of the photocurrent in a short time scale (<10 μs) could be measured.

## 3. Results and Discussion

### 3.1. TFA imager

**Figure 3.**
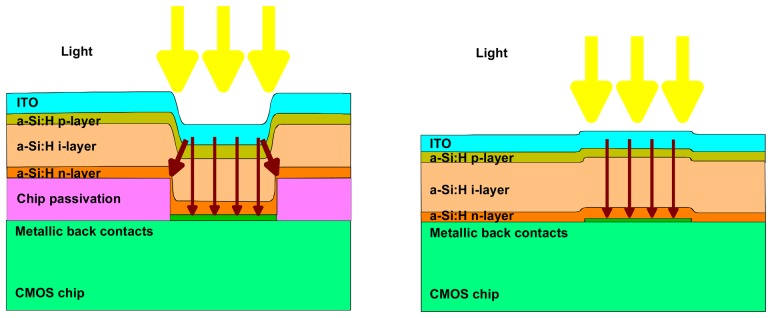
Schematic side view of TFA sensors pixels fabricated using passivated chips (left) and unpassivated chips (right). Higher leakage current density usually takes place at the pixel periphery due to higher defect density and higher electric field. In case of unpassivated chips, the n-layer offers a conduction path from the edge of the passivation to the metallic back contact.

Optimization of the fabrication process led to the fabrication of a-Si:H large are diodes (≥1 mm^2^) in various configuration n-i-p, p-i-n or metal-i-p with dark current leakage as low as 1 pA/cm^2^ for 1 μm thick diode at -1 V reverse bias [6]. However, non flat surface and the periphery of such diodes can significantly deteriorate the leakage current. This is especially critical in the case of TFA sensors where the a-Si:H diode array is deposited over the chips passivation layers. Opening in the latter to access the underneath metal layer which then form the back contact of each pixel diodes lead to a step in the a-Si:H layer and a strong increase in the leakage current (see Fig. 3) [6, 8].

To overcome such detrimental effect, metal-i-p diode configuration deposited on unpassivated chips has been used with success [6]. Nevertheless, it was essential to address also the effect on other characteristics of these imagers such as:

■ Fixed patterned noise FPN which characterizes the pixel-to-pixel variation in darkness
■ Photo response non uniformity PRNU which characterizes the pixel-to-pixel variation under illumination at half of saturation exposure. FPN and FPU are given by
  (1) $$\text{FPN or PRNU}=\sqrt{\frac{\sum_{i=1}^{n}{\left(x_{i}-x_{\text{mean}}\right)}^{2}}{n}}$$
  where *x_mean_* is the average signal on the whole array, i is the pixel index, n the pixel number and *x_i_* represents each individual pixel signal.

- Temporal noise TN given by
  (2) $$TN_{\text{rms}}=\frac{1}{\sqrt{2}}\sqrt{\frac{\sum_{i=1}^{n}{\left(x_{1i}-x_{2i}\;\right)}^{2}}{n}}$$
  where *x_1i_* is the signal of pixel *i* at time 1 *x_1i_* is the signal of pixel *I* at time 2.
- Radiometric sensitivity at a given wavelength *λ*
  (3) $$S\;(\lambda )=\frac{A_{\text{eff}}}{C_{\text{eff}}}\frac{\lambda q}{hc}\eta \;(\lambda )$$
  where *A_eff_* is the effective amplification factor, *C_eff_* the effective capacitance of the pixel, *h* the Planck constant, *c* the speed of light *q* the elementary charge, *λ* the wavelength and *η* the quantum efficiency.
- Dynamic range DR given by the maximum signal (voltage swing value) divided by the rms noise (here the temporal noise)
  (4) $$DR=20\log\left(\frac{V_{\max}}{V_{\text{noise}}}\right)$$

The performance of several imagers and a comparison with the naked CMOS readout chip are indicated in Table 1. The performance data were determined in the annealed state (after thermal annealing at 180°C for 1h30) and all defective pixels (shunted and/or showing no photo response) were discarded. The introduction of a metal-i-p structure (by removing the n-layer of a n-i-p structure) suppresses the lateral charge collection from the metal contact (see also Fig. 3). The pixel diode area is then strictly defined by the metal pad area and this leads to a marked improvement of the pixel uniformity (as characterized by FPN and PRNU) as well as for the temporal noise. However, the suppression of the n-layer also reduces the internal electrical field which reduces the sensitivity and the dynamic range.

By depositing the diode array on unpassivated chips, surface morphology is smoother leading to a situation (with regards to the layer growth) very close to test structure on glass. Peripheral effects are minimized leading to low dark current and FPN and TN very close to the one measured on a naked CMOS readout chip; homogeneity and TN of the a-Si:H diode array is found to at least as good as the CMOS chip, or of a CMOS imager based on that technology. As observed, the performance strongly depends on the chip surface morphology. The unpassivated chips used in this study still show significant surface roughness with marked steps at the periphery of the metal contact. By using chips with flatter surface, even better performance could be attained. Regarding dark current, values as low as 12 pA/cm^2^ could be measured on single 38×38 μm^2^ pixels at (room temperature), at reverse bias of -1 V (in the annealed state) [6].

**Figure 4.**
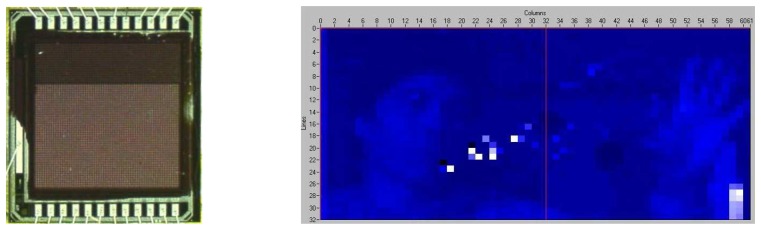
Picture of a TFA imager and corresponding image taken by this imager. This imager has 64×64 pixels, but only 32×64 were available for imaging. This other half of the chip was used for circuit development and testing.

Figure 4 shows a picture of a TFA imager fabricated on an unpassivated chip with an image taken out with such device. The defects seen in the image are due to defects on the CMOS chips created during the dicing of such unpassivated chips. Due to the use of chips from MPW, no action could be undertaken to improve the chip quality.

Light-induced degradation of the photodiodes is expected to affect the imager performance. Basically, it is going to increase mainly the dark current as it will be discussed in the next section. As long as the photodiodes are sufficiently reverse biased, no significant drop in the photogenerated carrier collection should occur. The main affected characteristic will therefore be the FPN especially if only part of the array is strongly illuminated for a long time (and therefore degraded).

### 3.2. Light induced degradation and dark current

**Figure 5.**
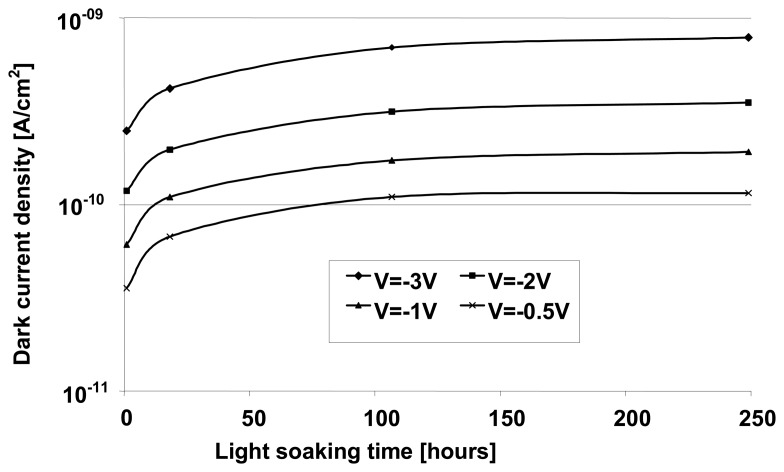
Dark current density of a TFA imager pixel (in the metal-i-p configuration) as a function of light-soaking time and pixel diode reverse bias voltage. Light-soaking conditions were AM1.5 white light at 100 mW/cm^2^ and 50°C.

Light-induced degradation of a-Si:H leads to an increase of the defect density (density of Si dangling bonds). Such increase will have a direct impact on the magnitude of the dark current as the density of thermally generated current depends (in steady-state) on the defect density and the bandgap of the semiconductor material [6, 12]:

(5) $$J_{th}=qd_{i}N_{db}kT\omega_{0}\;\exp\left[-E_{G}/2kT\right]$$

where *kT* is the temperature and Boltzmann constant product, *d_i_* the intrinsic layer thickness, *N_db_* the dangling bong density, *E_G_* the mobility band gap, and *ω_0_*∼10^13^ s^-1^ is the excitation rate prefactor (or attempt to escape frequency). Note that we assume here that the Fermi level position is located at mid-gap. Defect created upon light-soaking are meta-stable and can be annealed out. The density of these defects therefore depends both on the light intensity and temperature and on the history of light-illumination and temperature of the pixel. For standard light-soaking conditions as used for solar cells (full sun illumination at 50°C) a stabilization is observed after roughly 100 hours of light soaking leading to an increase in dark current by a factor of 2 to 3 depending on the polarization voltage (see Fig. 5).

### 3.3. Dark current decay

**Figure 6.**
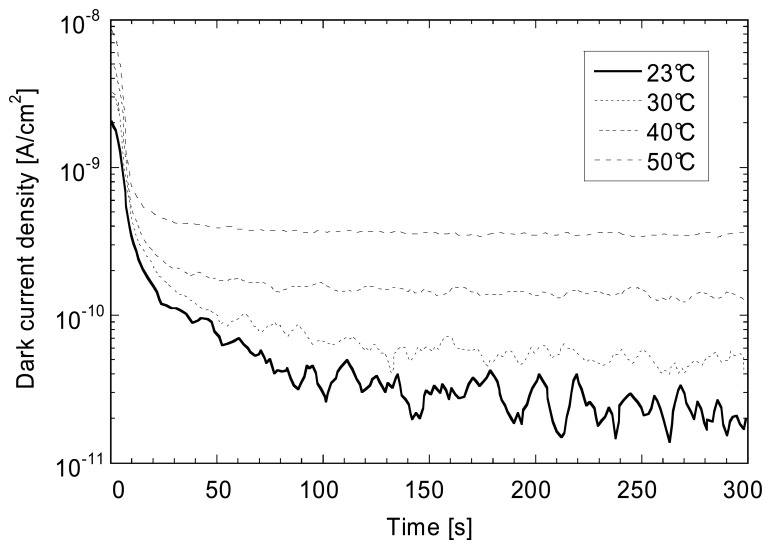
Dark current decay as a function of temperature of a 4 mm^2^ 0.88 μm thick n-i-p test diode after switching on a -3 V reverse bias voltage.

The continuous distribution of localized state which is present in the pseudo-gap of a-Si:H (see Fig. 2) is playing the role of charge reservoir and will either empty or fill-up as the concentration of free carriers present in the conduction band and valence band change. Increasing the reverse bias voltage will deplete those states. The time needed for reaching a state-state value for the dark current J_dark_ will depend on temperature as given by Eq. 5. As observed in Fig. 6 for 0.88 μm thick n-i-p diode, steady-state *J_dark_* values are attained after 200-300 s at room temperature after switching on the reverse bias voltage.

### 3.4. Photocurrent decay

A comparable transient behavior is observed after switching off (or reducing the light intensity) the light. Steady-state condition is again reached by thermal generation of carriers out of the localized states. For electrons, all carriers trapped at the level of the quasi Fermi level $E_{F}^{n}\left(t\right)$ will be released at time $\tau_{\text{rel}}^{n}$ [12]:

(7) $$\tau_{\text{rel}}^{n}=\frac{1}{\omega_{0}}\exp\frac{E_{C}-E_{F}^{n}\left(t\right)}{kT}$$

A similar expression is obtained for hole release.

**Figure 7.**
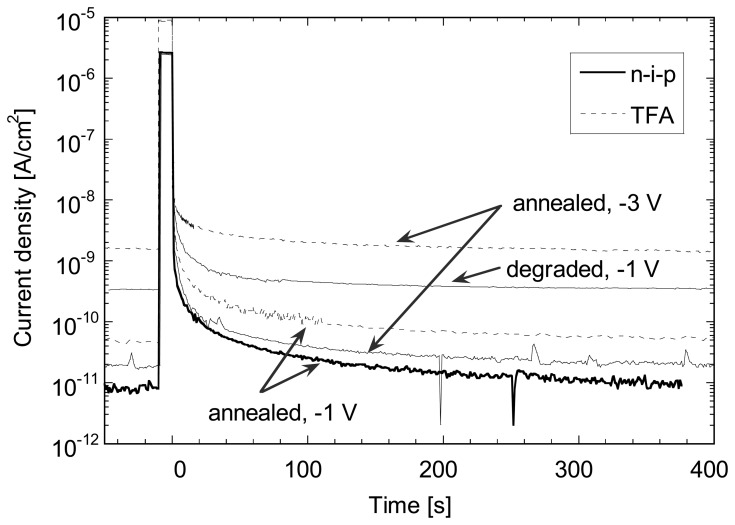
Comparison of current after a light pulse of 10 s (from a red LED, λ=650 nm, ≈10 μW/cm^2^), or photocurrent decay, for different bias voltage in the annealed state and after 1000 h of light soaking at 50°C for 1 μm thick n-i-p diode (with an area of 4 mm^2^) deposited as well as for corresponding pixel diodes of TFA imager (in metal-i-p configuration).

On Fig. 7 one can observe the evolution of the photocurrent after a light pulse of 10 s emitted by a red LED as measured on a 1 μm thick n-i-p diode deposited on glass and on a corresponding TFA pixel diode. We can notice that after a fast decrease of the current, first few seconds after the end of the pulse, the transient photocurrent decreases slowly by about one order of magnitude over a period of approximately 300 s reaching *J_dark_* values close to the steady-state values; the latter is given by the current density before the light pulse. This decay time is, as expected, close to the on observed for dark current decay. The time needed to empty all traps is then given by the position of the dark Fermi level position. Assuming a position of *E_C_*-*E_F_*≈0.9 eV, we can estimate a decay time of >100 s in agreement with our measurements. Note that the decay is non-exponential because the quasi-Fermi energy level is time dependent and the current strongly depends on the localized states distribution.

**Figure 8.**
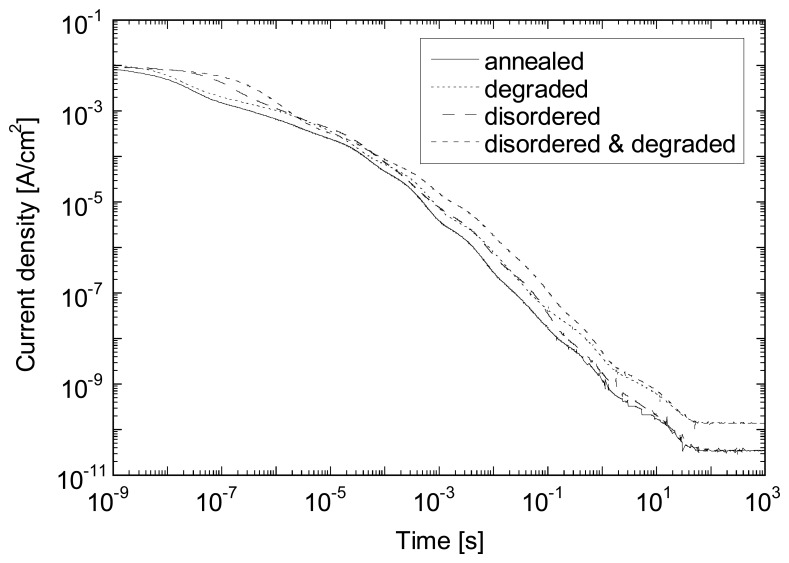
Simulated photocurrent decay for several 1 μm thick n-i-p diodes following the switch off of the light (AM1.5 white light, 100 mW/cm^2^) at -1 V. Diodes include either a state-of-the-art intrinsic a-Si:H layer or a strongly disordered material, both in the annealed state or in the light-soaked state (see text for details).

No important influence of the bias voltage for the decay time can be observed, although *J_dark_* (-3 V) increases by a factor of about 2 compared to *J_dark_* (-1 V) for test diodes and here more than 1 order of magnitude for TFA pixel diode. This dependence of *J_dark_* on bias voltage may vary from sample to sample (see also Fig. 5) especially when the dark current is controlled by peripheral effect (e.g. due to the surface morphology of the substrate). Here, the small pixel size (38×38 μm^2^) and the non planar configuration of the pixel are responsible for this leakage increase due to a local increase of the electric field as well as an increase of the defect density at the edges and corners of the pixels [6, 8]. The large increase of *J_dark_* shows that this additional leakage is highly sensitive to the applied bias voltage and becomes the largest contribution to *J_dark_* for reverse bias higher than -1 V. A similar behavior (no change in the decay time constant but an increase of the steady-state *J_dark_* value is observed upon light-soaking for test diodes. As *J_dark_* is mainly controlled by the density of defects, one can conclude that the latter has no or limited influence on the photocurrent decay time. Note that, as photocurrent does not decay exponentially in a-Si:H (due to the presence of a continuous state distribution in the bandgap) [12], it cannot be characterized by a single time constant. However, such decay can be fitted by an exponential (and its related time constant) over a restricted range of photocurrent decrease. These various time constants appear (see also Fig. 8) to be quite independent from the defect density value.

Figure 8 exhibits simulated photocurrent decay of several 1 μm thick n-i-p diodes following the switch off of the light (AM1.5 white light). Simulations have been performed using SC-SIMUL [13] for a diode with state-of-the-art a-Si:H intrinsic material, a diode with a strongly disordered material (characteristic energy of the slope of the band tail state distribution increased by 40%) both in the annealed state or in the light-soaked state (density of state increased from 5×10^15^ to 5×10^16^ cm^-3^). The SC-SIMUL program comprises a comprehensive description of localized states in a-Si:H. Thereby the amphoteric nature of dangling bonds is considered rather than using simpler Schockley-Read description statistics. As we can observe, effect of the wider band tails is limited to time below 10 s, while the effect of defect states for time bigger than 1 μs. In between those 2 time boundaries, both are playing a role. The time needed for reaching steady-state condition is, as observe experimentally, of the order of 200 s. One can also notice that neither the band tails nor the defect density affects the photocurrent decay time, as also observed experimentally at least between annealed and light-soaked samples.

**Figure 9.**
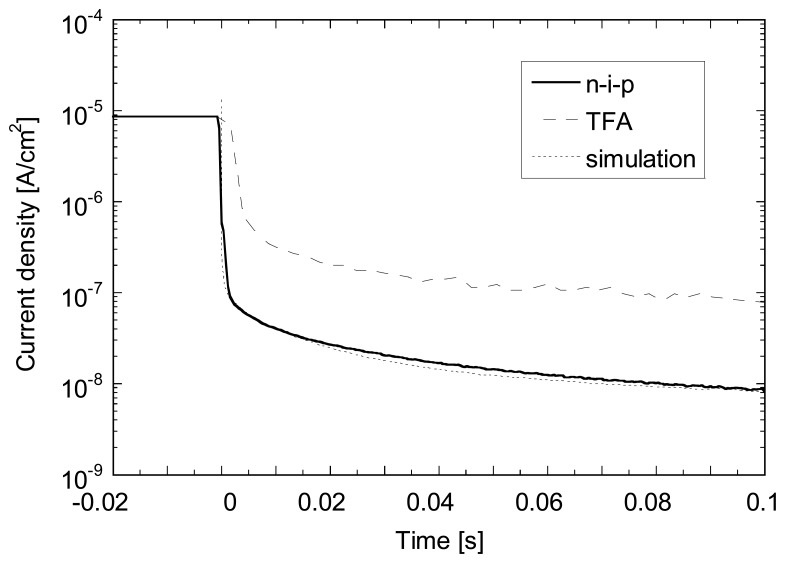
Detail of the first 100 ms after the switch off of the light (red LED, λ=650 nm, ≈10 μW/cm^2^) for the same devices presented in Fig. 2 and the corresponding simulated photocurrent decay following the switch off of the light (white-light adjusted to give approximately the same experimental photocurrent) at -1 V.

Details of the photocurrent decays (as shown in Fig. 7) are plotted in Fig. 9, together with a simulated curve. For the latter, the illumination condition was adjusted to achieve the same photocurrent as the experimental one during the light pulse. As we can observe an almost perfect match is obtained between the experimental photocurrent decay measured on a n-i-p test diode and the simulated curve. The photocurrent decay for the TFA device shows very similar time constants (despite the match higher current value) except for the first 30 ms where the decay is significantly slower. This difference is not yet well understood, but might be linked to charge release at the periphery of the pixel that contributes to a higher current.

## 4. Experimental Conclusions

In this work, we have analyzed the performance of a TFA image sensor. Transient current behavior of the TFA pixel diodes as well as corresponding test diodes deposited on glass have been measured and compared. Even though a-Si:H diodes are well suited for high dynamic range imaging and low light level detection, the long photocurrent decay after an illumination limits the time before a new acquisition or can limit the dynamic. The time constants for these long decays are demonstrated to be rather independent on both the band tails states and defect density of a-Si:H.

Both the current density during the decay after an illumination (or a change of polarisation of the diode) and the steady-state dark current are, in TFA devices, strongly controlled by peripheral effects and therefore exhibit higher values than observed in corresponding test diodes deposited on glass. Note here that light-soaking (which leads to an increase of the defect density) has here a more detrimental effect on diodes on glass than on TFA devices as, for the latter, the light-soaking effect is partially masked by the peripheral effect on dark current.

This slow current decay leads to the observation of image lag in TFA imagers. This feature appears to be intrinsic to the use of a-Si:H, as the current decay time does not depend on the defect density. A limit in the frequency operation of the device is then given mainly by the required sensitivity or dynamic range. The latter can be very high when the sensor is operated at very low frequency.

In TFA sensors with small pixels, complexity of the circuit has to be reduced. In this case a simple 3 transistors circuit is generally used and the charge integration is done by the pixel diode. This leads to change in the bias voltage and further transient effect during the exposition as well as during reset of the diode. In this configuration dependence of image lag on defect density is also observed [14].

Nevertheless, we have demonstrated that image sensors with high sensitivity, high dynamic, low dark current and low noise can be fabricated using TFA technology. Despite the intrinsic limitations introduce by the gap states of a-Si:H, this technology is expected to offer interesting performance for low speed and high sensitivity applications.

## Figures and Tables

**Table 1.** Overview of several TFA sensors performance using either a metal-i-p or n-i-p diode configuration deposited on passivated or unpassivated chips. Some values measured on the naked CMOS readout chips are also indicated for comparison purposes.

Performances	naked CMOS chip	metal-i-p on unpassivated chip	metal-i-p on passivated	n-i-p on passivated chip
Dark current @-1V		49.7 pA/cm2	486 pA/cm2	2600 pA/cm2
FPN	5.30 mV	5.13 mV	5.73 mV	63.25 mV
PRNU		6.9 mV	7.82 mV	125.4 mV
Temporal Noise	0.97mV	1.08 mV	1.73 mV	6.11 mV
Sensitivity @ 600nm & -2V		56 V/μJ cm-2	51 V/μJ cm-2	70 V/μJ cm-2
Dynamic Range		60.7 dB	56.6 dB	45.7 dB
